# Neonatal Mortality and Associated Factors Among Neonates Admitted to the Neonatal Intensive Care Unit of Dil Chora Referral Hospital, Dire Dawa City, Ethiopia, 2021: A Facility-Based Study

**DOI:** 10.3389/fped.2021.793160

**Published:** 2022-02-11

**Authors:** Gelila Thomas, Melake Demena, Behailu Hawulte, Addis Eyeberu, Helina Heluf, Dawit Tamiru

**Affiliations:** ^1^Department of Obstetrics and Gynecology, Dil Chora Referral Hospital, Dire Dawa, Ethiopia; ^2^School of Public Health, College of Health and Medical Sciences, Haramaya University, Harar, Ethiopia; ^3^School of Nursing and Midwifery, College of Health and Medical Sciences, Haramaya University, Harar, Ethiopia

**Keywords:** neonate, NICU, newborn, perinatal morbidity, neonatal mortality, Dire Dawa

## Abstract

**Background:**

Despite the incredible progress made in decreasing under-five mortality, neonatal mortality remains the main and slowly advancing contributor. Though all efforts were made to decline the death of a newborn, current information showed that newborn death is unaverted and not a fastened agenda. This study aimed to assess neonatal mortality and its determinants among neonates admitted at the Dil Chora Hospital in Dire Dawa City.

**Methods:**

Facility-based cross-sectional study design was employed. A total of 376 newborns were selected systematically from neonates admitted to the NICU of the Dil Chora Referral Hospital from June 1, 2017 to December 31, 2020. Data were collected from medical records using a standard extraction checklist. The data were entered into Epi-data version 3.1 and then exported into SPSS version 24 for statistical analysis. Bivariate and multivariate analyses were employed to identify the association between independent variables and neonatal death.

**Result:**

The prevalence of neonatal death was 11.4% (95% CI: 9.44, 13.36). The majority of 37 (86.05%) of the neonates died within 7 days of life. The most common causes of admission included perinatal asphyxia (55.3%), hypoglycemia (21.5%), and hypothermia. Nearly half (40.4%) of the mothers of newborns experience index pregnancy complications, premature rupture of the membrane (AOR = 5.79, 95% CI: 2.08, 16.1), birth weight <2,500 g (AOR = 3.96, 95% CI: 1.56. 10.06), hypothermia (AOR = 2.54, 95% CI: 1.1, 6.02), index pregnancy complications (AOR = 4.79, 95% CI: 1.92, 11.91), and induced labor (AOR = 4.45, 95% CI: 1.53, 12.94), which were significantly associated with neonatal mortality.

**Conclusion:**

The prevalence of neonatal mortality was high compared with the national target. Premature rupture of the membrane, birth weight <2,500 g, hypothermia, index pregnancy complications, and induced labor were significantly associated with neonatal mortality. The majority of neonatal deaths are due to complications arising from pregnancy, labor, and delivery, and lack of quality of care at the neonatal intensive care unit. Cultivating and increasing the utilization of antenatal care services, quality of care at delivery, and the neonatal intensive care unit could avert those deaths.

## Introduction

The first 28 days of life is an extremely susceptible time for a newborn to complete many of the physiologic adjustments required for life outside the uterus, which makes it the most dangerous period in life (1, 2). Neonates in need of serious medical attention are admitted to the neonatal intensive care unit (NICU), which incorporates innovative technology and trained staff to effectively provide specialized care for neonates (3).

Despite the incredible progress made in decreasing under-five mortality, neonatal mortality remains the main and slowly advancing contributor. From 5.3 million under-five deaths, around 47% of deaths occurred in the neonatal period (4). It is urgently required to accelerate the progress of achieving target 3.2 of the sustainable development goals (SDGs), which calls for an accelerated and coordinated continuum of care (5). Unless the progress is fast-tracked, 26 million newborns would die from 2019 to 2030 (4).

Though all efforts were made to decline the deaths of the newborn, current information showed that newborn death is unaverted and not a fastened agenda. An Ethiopia Mini-Demographic Health Survey (Mini-EDHS) report showed that there is an increment in neonatal mortality from 28 in 2016 to 30 per 1,000 live births in 2019 (6). The majority of newborn deaths occur in the early neonatal period, which is a critical time for prevention, control, and monitoring complications arising from the delivery (6). Evidence showed that 70% of the current neonatal deaths could be prevented through the practice of recommended neonatal care practices as it is recognized as an excellent solution to reduce the mortality of newborn babies (7).

Ethiopia missed the target of the national newborn and child survival strategy and is in danger of missing the SDGs target 3.2 on newborn deaths (4). Therefore, it is a critical time to maximize a wide-ranging way of execution of strategies to decrease neonatal death. Furthermore, it is recommended to continuously assess and monitor neonatal health. In addition, it is important to identify the cause and determinant factors of death, which is fundamental for the prevention of newborn death and monitoring public health strategies (8). Different studies were conducted in assessing the prevalence of neonatal mortality, but the perinatal health conditions of neonates were not assessed as they contribute to the causes of neonatal mortality. Even though Ethiopia lies among the highest level of neonatal mortality rate compared with other sub-Saharan African countries, only little is known about the magnitude and related factors of neonatal death. So, the study aimed to assess the magnitude and determinants of neonatal mortality at the Dil Chora Referral Hospital in Dire Dawa City, Ethiopia.

## Methods And Materials

### Study Design, Setting, and Period

A facility-based cross-sectional study was conducted in the neonatal intensive care unit (NICU) of the Dil Chora Referral Hospital in Dire Dawa City administration. Dire Dawa City is located 515 km toward the east of the capital Addis Ababa. The city is one of the two chartered cities in Ethiopia and has 5 hospitals (2 public and 3 private), 17 health centers, and 34 health posts. The Dil Chora Referral Hospital provides both general and specialist services for the population of eastern Ethiopia with more than 3,000 deliveries per annum. The admission criteria to the NICU of the Dil Chora Referral Hospital include high-risk neonates delivered within the institution and referrals from other health facilities, neonates having neonatal complications, and premature neonates. A neonatal intensive care unit (NICU) equipped with essential equipment for caring for neonates with special care needs is available. It includes incubators, radiant warmers, phototherapy machines, infusers, oxygen cylinders, pulse oximetry, glucometer, and neonatal resuscitation equipment. The unit is staffed with nurses, pediatricians, and pediatric residents. The neonates of the study recruits were admitted to the NICU from June 1, 2017 to December 31, 2020.

### Study Population

All systematically selected neonates who were admitted to the NICU of the Dil Chora Referral hospital were the study population. Medical records with incomplete information for the outcome variables, leave against medical advice, and referred to other facilities were not included in the study.

### Sample Size Determination and Sampling Procedure

The sample size was calculated by using a single population proportion formula with assumptions of confidence level at 95% = 1.96, a margin of error (d) = 0.045, and magnitude of neonatal death (*p* = 0.23) from a previous study conducted at the NICU of Gondar Referral Hospital (9). By adding 13% for incompleteness, the final sample size became 381. The study participants were selected by systematic random sampling method by using the registration number of the neonates. Among a total of 1,300 neonatal registration numbers, every three participants were selected. The first study unit was selected using the lottery method.

### Data Collection Methods and Quality Control

The data were collected by using a data extraction checklist adapted and modified from diverse works of literature (10–14). The data collection tool was pretested at the Haramaya Hospital. Using the data collection tool, sociodemographic characteristics of the mother, complications during the pregnancy, place of delivery, gestational age, age of the neonate, and length of stay were collected. The data were collected by five well-trained BSc-holding nurses. The investigators checked the collected data on a daily basis for consistency and completeness.

### Operational Definition

Neonatal mortality is the death of the newborn after admission to the NICU and before discharge, and confirmed and recorded on the chart.

Antenatal care visit is any history of visit or follow-up during the current or index pregnancy at any health institution for a checkup of pregnancy and designated or recorded on a chart.

Intrapartum complications are complications that occurred after the onset of labor including intrapartum bleeding, obstructed labor, prolonged labor, eclampsia, chorioamnionitis, and others.

Congenital malformation is body deformity or deformities from birth that are believed to have an impact on the health of the baby, diagnosed and recorded on charts by professionals on admission.

Hypoglycemia is a measure of low blood glucose (40 mg/dl) that was diagnosed and recorded on charts by professionals on admission (10).

Hypothermia is a low-body temperature measurement (<36.5°C), and is diagnosed and recorded on charts during the admission of neonates (11).

Birth asphyxia is diagnosed whenever a neonate had an Apgar score of <6 in the 5th min and/or if he/she did not cry immediately after birth, had respiratory distress, floppiness, loss of consciousness, presence of convulsion, and loss of neonatal reflexes (10).

Birth weight is classified using the WHO weight classification, very low birth weight is any child with birth weight <1,500 g, while low birth weight is any child with birth weight <2,500 g (15).

Premature rupture of membranes (PROM) is the spontaneous leakage of amniotic fluid from the amniotic sac, occurring after 28 weeks of gestation, and before the onset of labor, and was diagnosed and recorded on charts by professionals on admission.

### Statistical Analysis

The collected data were coded and entered into EPI data version 3.1 and then exported to SPSS version 24 statistical packages software for cleaning and analyses. Data were summarized using frequency distributions, tables, and figures. Bivariate binary logistic regression analysis was carried out to observe the association between each independent variable and the outcome variable. All variables with a value of *p* < 0.25 during bivariate analyses were considered for multivariable logistic regression analysis. To control the possible confounders and to identify factors associated with the outcome variable, multivariate analysis was carried out. The odds ratio along with the 95% confidence interval was estimated to measure the strength of the association. All the assumptions of regression analysis (model adequacy and multicolinearity of independent variables) were checked. The level of statistical significance was declared at a value of *p* < 0.05.

## Result

### Sociodemographic Characteristics of the Mothers and Neonates

Out of 381 selected neonates, five neonates had incomplete medical records. A total of 376 neonates were included in the study. Almost two-thirds (65.4%) of the neonates were males, and more than one-fourth (27.4%) came from rural areas. The majority (38.8%) of the mothers of neonates were between 25 and 29 years old with a median age of 27 years with an interquartile range of 7 years (Table 1).

**Table 1:** Sociodemographic characteristics of the neonates and mothers of the neonates admitted at the neonate intensive care unit (NICU) of the Dil Chora Referral Hospital, Eastern Ethiopia, 2021.

**Variable**	**Categories**	**Neonatal death**		***p*-value**
		**Yes**	**No**	
Sex	Female	18 (13.8%)	112 (86.2%)	Ref.
	Male	25 (10.2%)	221 (89.8%)	0.288
Residency	Urban	15 (5.5)	258 (94.5)	Ref.
	Rural	28 (27.2)	75 (72.8)	0.001
Age of neonates	<1 day	40 (12.7)	274 (87.3)	Ref.
at admission	≥12 days	3 (4.9)	59 (95.1)	0.087
Maternal age	<20	8 (29.6)	19 (70.4)	Ref.
in years	20–24	13 (15.3)	72 (84.7)	0.692
	25–29	9 (6.2)	137 (93.8)	0.150
	30–34	7 (90.1)	64 (9.9)	0.622

*Ref., reference. The model used was bivariate binary logistic regression*.

### Perinatal Characteristics of Neonates

The mean length of stay in the NICU was 5.7 (SD ± 4.7) days. One hundred twenty-six (33.5 %) of the neonates had very low birth weight. More than half, 208 (55.3%), of the neonates had perinatal asphyxia, while 81 (21.5%) of the neonates had hypoglycemia. Nearly three quarters (72.8%) of the newborns were delivered after 37 completed weeks of gestation, and 327 (87%) were singletons. Related to breastfeeding initiation, the majority (80.9%) of the mothers did not start breastfeeding within the first hour of delivery (Table 2).

**Table 2:** Perinatal characteristics of neonates admitted to the NICU of Dil Chora Referral Hospital, Eastern Ethiopia, 2021.

**Variable**	**Categories**	**Neonatal death**		***p*-value**
		**Yes**	**No**	
Birth weight in grams	≥2,500	11 (4.4)	239 (95.6)	Ref.
	<2,500	32 (25.4)	94 (74.6)	0.001
Breastfeeding initiated	Yes	2 (2.8)	70 (97.2)	Ref.
within 1 h	No	41 (13.5)	263 (86.5)	0.021
Temperature at	≥36	25 (8.5)	270 (91.5)	Ref.
admission in °C	<36	18 (22.2)	63 (77.8)	0.001
Hypoglycemia	No	25 (8.5)	270 (91.5)	Ref.
	Yes	18 (22.2)	63 (77.8)	0.296
Perinatal asphyxia	No	19 (11.3)	149 (88.7)	Ref.
	Yes	24 (11.5)	184 (88.5)	0.045
Newborn resuscitation	Yes	21 (15.8)	112 (84.2)	Ref.
at birth	No	22 (9.1)	221 (90.9)	0.052
Gestational age of	>42	10 (4.4)	218 (95.6)	Ref.
neonates in weeks	37–42	1 (4.5)	21 (95.5)	0.314
	<37	32 (25.4)	94 (74.6)	0.485
Fetal presentation	Cephalic	39 (11.4)	304 (88.6)	Ref.
	Others	4 (12)	29 (88)	0.848
Congenital anomalies	No	41 (11.4%)	320 (88.6%)	Ref.
	Yes	2 (13.3%)	13 (86.7%)	0.814

### Maternal and Obstetric Characteristics

Three hundred thirty (87.8%) of the mothers of the newborns had at least one ANC follow-up. About half (51.8%) of the mothers were multipara. One hundred fifty-two (40.4%) of the mothers of the newborns experienced index pregnancy complications. The type of complications includes preeclampsia (32), eclampsia (16), diabetes mellitus (3), antepartum hemorrhage (27), poly/oligohydramnios (29), chorioamnionitis (20), and others (cardiac disease, obstructed labor) (25). Regarding delivery, 294 (78.2%) of the mother had a spontaneous onset of delivery, and 373 (99.2%) of the mothers delivered their newborns at health institutions. About 151 (40.2%) of the mothers of the neonates had an intrapartum complication, and 198 (52.7%) had premature rupture of membrane before the onset of labor (Table 3).

**Table 3:** Maternal obstetric characteristics of neonates admitted to the NICU of Dil Chora Referral Hospital, Eastern Ethiopia, 2021.

**Variable**	**Categories**	**Neonatal death**		***p*-value**
		**Yes**	**No**	
Parity	Above 4	7 (17.5)	33 (82.5)	Ref.
	2–4	11 (7.1)	144 (92.9)	0.050
	<2	25 (13.8)	156 (86.2)	0.550
Antenatal care	Yes	21 (6.4)	309 (93.6)	Ref.
(ANC)	No	22 (47.8)	24 (52.2)	0.001
Type of pregnancy	Singleton	36 (11%)	291 (89%)	Ref.
	Multiple	7 (14.3%)	42 (85.7%)	0.503
Place of delivery	Health institution	41 (11)	332 (89)	Ref.
	Home	2 (66.7)	1 (33.3)	0.024
Index pregnancy	No	8 (4)	216 (96)	Ref.
complication	Yes	35 (22.5)	117 (77.5)	0.0001
Onset of labor	Spontaneous	28 (9.5)	266 (90.5)	Ref.
	Induced	15 (18.3)	67 (81.7)	0.03
Premature rupture	No	11 (6.2)	167 (93.8)	Ref.
of membrane (PROM)	Yes	32 (16.2)	166 (83.8)	0.003
Duration of PROM	<12 h	14 (10.52)	119 (89.48)	Ref.
	≥12 h	18 (27.8)	47 (72.2)	0.003
Birth trauma at birth	No	37 (11.1%)	297 (88.9%)	Ref.
	Yes	6 (14.3%)	36 (85.7%)	0.539

### Prevalence of Neonatal Mortality

Among newborns admitted to the NICU of Dil Chora Referral Hospital, the majority, 333 (88.6 %) of the newborns were alive, while 43 (11.4 %) of the newborns died before discharge. Therefore, the prevalence of neonatal death was 11.4% (95% CI: 9.44–13.36). The majority, 37 (86.05%), of the neonates died within 7 days of life, while six (3.95) died between 7 and 28 days of life. The most common causes of neonatal death were perinatal asphyxia 24 (55.8%), low birth weight 32 (74.1%), hypothermia 18 (41.8%), hypoglycemia 18 (41.8%), and multiorgan failure 16 (37%).

### Factors Associated With Neonatal Mortality

Variables including residence, antenatal care visit at least once, birth weight, the onset of labor, hypothermia, PROM, newborn resuscitation, and complication during pregnancy were considered for multivariate logistic regression analysis to control the effect of confounders. Variables including antenatal care visits at least once, birth weight, the onset of labor, hypothermia, PROM, and complication during pregnancy were significantly associated with neonatal mortality in multivariable logistic regression analysis.

Neonatal mortality was 4.11 times more likely (AOR = 4.11; 95% CI: 1.53, 11.03) to occur among women who did not attend ANC visits at least once compared with their counterparts. The odds of neonatal mortality were 2.54 and 3.96 times more likely to occur among newborns who had hypothermia and low birth weight than neonates without the conditions (AOR = 2.5; 95%CI: 1.1, 6.02) (AOR = 3.96; 95%CI: 1.56, 10.06), respectively. In multivariate analysis, neonatal mortality was about 4.79 times (AOR = 4.8; 95%CI: 1.9, 11.9) more likely in mothers who had a complication during their last pregnancy compared with mothers who had no pregnancy complication. The odds of neonatal mortality were about 5.79 times higher in neonates whose mothers experienced PROM compared with those who had no PROM (AOR = 5.79, 95%CI: 2.08, 16.1). The induced onset of labor was about 4.45 times more likely to increase the chance of neonatal mortality compared with those mothers who had spontaneous onset of labor (AOR = 4.5, 95%CI: 1.5, 12.9) (Table 4).

**Table 4:** Determinants of neonatal mortality at the NICU of Dil Chora Referral Hospital, Eastern Ethiopia, 2021.

**Variable**	**Categories**	**Neonatal death**		**COR**	**AOR**	***p*-value**
		**Yes**	**No**			
Residency	Rural	28 (27.2)	75 (72.8)	6.42 (3.26–12.64)	2.18 (0.92, 5.21)	0.078
	Urban	15 (5.5)	258 (94.5)	1	1	Ref.
ANC	No	22 (47.8)	24 (52.2)	13.48 (6.51–27.93)	4.11 (1.53, 11.03)	0.001^*^
	Yes	21 (6.4)	309 (93.6)	1	1	Ref.
PROM	Yes	32 (16.2)	166 (83.8)	2.92 (1.42–6.00)	5.79 (2.08, 16.1)	0.001^*^
	No	11 (6.2)	167 (93.8)	1	1	Ref.
Hypothermia	Yes	18 (22.2)	63 (77.8)	3.08 (1.58–6.00)	2.54 (1.1, 6.02)	**0.035**
	No	25 (8.5)	270 (91.5)	1	1	Ref.
Birth weight	<2,500	32 (25.4)	94 (74.6)	0.135 (0.65, 0.278)	3.96 (1.56. 10.1)	**0.004**
	≥2,500	11 (4.4)	239 (95.6)	1	**1**	Ref.
Newborn resuscitation at birth	Yes	21 (15.8)	112 (84.2)	1.88 (0.99–3.57)	2.19 (0.93, 5.17)	0.075
	No	22 (9.1)	221 (90.9)	1	1	Ref.
Index pregnancy complication	Yes	35 (22.5)	117 (77.5)	8.07 (3.21–14.97)	4.79 (1.92, 11.9)	0.001^*^
	No	8 (4)	216 (96)	1		Ref.
Onset of labor	Induced	15 (18.3)	67 (81.7)	2.12 (1.07–4.28)	4.45 (1.53, 12.9)	0.005^*^
	Spontaneous	28 (9.5)	266 (90.5)	1		Ref.

* *p < 0.05. AOR, adjusted odds ratio; COR, crude odds ratio. Bold values indicates the variables that was significantly associated with the outcome variable*.

## Discussion

The majority of neonatal mortality in the developing world occurs as a result of poor quality of care during the antenatal, intrapartum, and postpartum periods. Since neonatal mortality has not declined, evidence suggested that continuous assessment is needed. Therefore, this study was intended to assess the prevalence and determinant factors of neonatal mortality at the Dil Chora Referral Hospital, Dire Dawa.

The magnitude of newborn death in the current study was 11.4% (95% CI:9.44–13.36), which was found to be consistent with a study done in Jimma University Medical Center, Southwest Ethiopia at 13.3% (16). However, the finding was lower compared with studies conducted in Ghana at 20.2% (17); Mizan Tepi University Teaching Hospital, Southwest Ethiopia, at 22.8% (11); Hiwot Fana Specialized University Hospital, Eastern Ethiopia, at 14.3% (18); and Gondar Referral Hospital, Northwest Ethiopia, at 23.1% (9). However, the prevalence of neonatal death was higher than studies conducted in the Somali Region, Ethiopia, at 5.7% (19); Gondar, Ethiopia, at 4.4% (12); and in Mekelle, Ethiopia, at 6.6% (20). The discrepancy might be due to the presence of sociocultural and socioeconomic differences across the Ethiopian regions. This variation might also be due to the variation in sample sizes (11, 17). Other possible justification may be the difference in the health service utilization including giving birth at health institutions by skilled care providers and health seeking for sick neonates, the variation in health institution setup (equipment available and skilled persons), and economic disparities among study participants (11, 13, 21). Besides, this study used data of those critically sick babies that were admitted to the neonatal intensive care unit.

This finding shows that newborns delivered from mothers who had pregnancy complications had a higher chance of death. This is comparable with a previous study conducted in the Tigray region, Northern Ethiopia (22). The likely explanation might be due to maternal health care during the antenatal period being a significant determinant of neonatal and maternal health. Moreover, pregnancy-related complications can adversely affect the wellbeing of neonates (23) based on the fact that neonates born from mothers with pregnancy complications were also in danger of being premature, having intrauterine growth restriction (IUGR), and respiratory distress syndrome (RDS) that lead neonates to death (24).

Neonates born from a mother with no ANC visit increases the odds of neonatal death compared with mothers with ANC visits. This finding is supported by a study conducted in the Afar region (21). Women who do not have antenatal care follow-up during pregnancy are more at a menace for pregnancy and intrapartum-related complications, which, in turn, place the newborn at danger of death (25). Therefore, women who complete antenatal care visits have a better chance of prompt detection and management of birth-related problems, and also establishing good health before childbirth and early postnatal period (21, 26). Besides, women who had complete ANC follow-up had an increased likelihood of giving birth by a skilled birth attendant who leads to having quality essential newborn care (ENC), which decreases the odds of neonatal death (16). Moreover, ANC provides an exceptional opportunity for health care providers to counsel mothers on how to recognize warning signs of complications during pregnancy, labor, and delivery. It is also an opportunity to encourage them to plan clean and safe deliveries at health institutions by trained attendants. Additionally, during ANC follow-up, health care providers can provide information regarding postpartum care, newborn care, a neonatal danger sign, danger signs of pregnancy, and appropriate actions to be taken (13).

This study found that neonates with birth weight <2,500 g were at higher odds of death than those having normal or higher birth weight. Other studies have consistently reported similar findings (16, 27, 28). The possible explanation is that low birth weight increases child susceptibility to infection and decreases their immune systems and other body defense mechanisms, which control newborn disease exposure. As a result, neonatal survival is decreased (29). Other possible explanations for this might also be due to the low-birth weight newborns are expected to suffer from hypothermia, infection, and poor immunological function, which increased the risk of neonatal deaths (16, 30).

The odds of death among hypothermic neonates were 2.5 times that of neonates who did not have the conditions. The possible justification is that hypothermia contributes to hypotension, hypoxia, hypoglycemia, bradycardia, disseminated intravascular coagulation, irregular and slow breathing, and shock (31, 32), which, in turn, increase long-term disability and death. Evidence showed that mortality was high among hypothermic neonates compared with normothermic neonates (33). A systematic review and meta-analysis pointed out that decreasing the risk of low birth weight, preterm, and neonates with neonatal problems is important for the prevention of the burdens of hypothermia (32).

This study revealed that neonates born from mothers who had PROM had a higher probability of newborn mortality than neonates born from mothers who did not have PROM. This is comparable with the study conducted in Jimma Specialized Hospital and Arba Minch General Hospital (16, 34). This might be due to PROM increasing the risk of acquiring neonatal sepsis, birth asphyxia, pulmonary hyperplasia, and preterm labor (35). Also, PROM is a risk factor of ascending infection to the uterine cavity, which results in the infection of the fetus (36).

This study revealed that the onset of labor is one determinant factor of neonatal death. The odds of neonatal death from mothers with induced labor were 4.5 times more likely to occur compared with those of spontaneous labor. Consistent findings were reported from a previous study done in Arba Minch General Hospital (37). The possible justification is the reason for induction. Usually, labor is induced if there are pregnancy complications, fetal abnormalities, and labor abnormalities. So, those reasons could contribute to the development of neonatal complications and finally result in neonatal death. The possible reason might be due to the medications provided to induce labor, which tend to cause abnormal or excessive contraction, which reduces the oxygen supply of the baby and causes fetal distress, and lowers the heart rate of the newborn (38).

The strength of the study was the utilization of a standard and pretested data extraction sheet. Again, the study provides the updated prevalence of neonatal mortality at the Dil Chora Hospital.

The limitation of this study is it might not show a cause–effect relationship because the study design was cross-sectional. The use of the medical records of newborns because of the incompleteness, and the study did not identify inborn and referrals may have an impact on the generalization.

## Conclusion

The prevalence of neonatal death was high compared with the national target. Factors such as having no ANC follow-up, induced labor, complications during pregnancy, PROM, hypothermia, and birth weight <2,500 g were statistically significant predictors of neonatal death. Majority of the neonatal deaths are resulting from complications arising from pregnancy, labor and delivery, and lack of quality of care at the neonatal intensive care unit. Cultivating the antenatal care services, early detection and treatment, and referral of high-risk pregnancies and newborns could avert newborn deaths.

## Data Availability Statement

The original contributions presented in the study are included in the article/supplementary material, further inquiries can be directed to the corresponding author/s.

## Ethics Statement

The studies involving human participants were reviewed and approved by Haramaya University, College of Health and Medical Sciences, Institutional Health Research Ethics Review Committee (IHRERC). The patients/participants provided their written informed consent to participate in this study.

## Author Contributions

GT conceived the idea of the study and had major roles in the data review, analysis, writing, and preparing of the manuscript. MD, BH, HH, AE, and DT played their role in reanalyzing, writing the final draft of the results, and wrote the manuscript. All authors were involved in reading and approving the final manuscript.

## Funding

Funding was provided by the Haramaya University.

## Conflict of Interest

The authors declare that the research was conducted in the absence of any commercial or financial relationships that could be construed as a potential conflict of interest.

## Publisher's Note

All claims expressed in this article are solely those of the authors and do not necessarily represent those of their affiliated organizations, or those of the publisher, the editors and the reviewers. Any product that may be evaluated in this article, or claim that may be made by its manufacturer, is not guaranteed or endorsed by the publisher.

## Abbreviations

ANC: antenatal care
EDHS: Ethiopian Demographic Health Survey
LBW: low birth weight
NICU: neonatal intensive care unit
SDGs: sustainable development goals
PROM: premature rupture of membrane
WHO: World Health Organization.
